# Investigating Differences in Vigilance Tactic Use within and between the Sexes in Eastern Grey Kangaroos

**DOI:** 10.1371/journal.pone.0044801

**Published:** 2012-09-12

**Authors:** Guillaume Rieucau, Pierrick Blanchard, Julien G. A. Martin, François-René Favreau, Anne W. Goldizen, Olivier Pays

**Affiliations:** 1 Institute of Marine Research, Bergen, Norway; 2 Université de Toulouse, CNRS, ENFA; UMR 5174, Laboratoire Evolution et Diversité Biologique, Toulouse, France; 3 Department of Ecology and Evolutionary Biology, University of California Los Angeles, Los Angeles, California, United States of America; 4 Laboratoire Biométrie et Biologie Evolutive, UMR5558 Centre National de la Recherche Scientifique (CNRS), Université Claude Bernard Lyon 1, Villeurbanne, France; 5 The University of Queensland, School of Biological Sciences, Queensland, Australia; 6 Groupe Écologie et Conservation, Université d’Angers, Campus Belle Beille, Angers, France; University of Manitoba, Canada

## Abstract

Aggregation is thought to enhance an animal’s security through effective predator detection and the dilution of risk. A decline in individual vigilance as group size increases is commonly reported in the literature and called the group size effect. However, to date, most of the research has only been directed toward examining whether this effect occurs at the population level. Few studies have explored the specific contributions of predator detection and risk dilution and the basis of individual differences in the use of vigilance tactics. We tested whether male and female (non-reproductive or with young) eastern grey kangaroos (*Macropus giganteus*) adopted different vigilance tactics when in mixed-sex groups and varied in their reliance on predator detection and/or risk dilution as group size changed. This species exhibits pronounced sexual dimorphism with females being much smaller than males, making them differentially vulnerable toward predators. We combined field observations with vigilance models describing the effects of detection and dilution on scanning rates as group size increased. We found that females with and without juveniles relied on predator detection and risk dilution, but the latter adjusted their vigilance to the proportion of females with juveniles within their group. Two models appeared to equally support the data for males suggesting that males, similarly to females, relied on predator detection and risk dilution but may also have adjusted their vigilance according to the proportion of mothers within their group. Differential vulnerability may cause sex differences in vigilance tactic use in this species. The presence of males within a group that do not, or only partially, contribute to predator detection and are less at risk may cause additional security costs to females. Our results call for reexamination of the classical view of the safety advantages of grouping to provide a more detailed functional interpretation of gregariousness.

## Introduction

Aggregation is commonly thought to provide security benefits to animals [1], [2] through a greater power of detection of predators (“the many eyes hypothesis” [3]) and the dilution of risk where the nearby presence of companions decreases the likelihood of any given individual being the victim of an attack [4], [5]. A well documented and taxonomically widespread effect is a decline in individual vigilance as animals’ group sizes increase: the group-size effect [6], [7]. Through the combination of improved collective vigilance and risk dilution, animals in large groups benefit from an enhanced safety that allows them to reallocate time saved in vigilance to other fitness-improving activities [8]. This reallocation might be crucial for prey species (including large mammalian herbivores) that are highly constrained by their food acquisition [9], [10]. In addition to its anti-predator component, vigilance may also be used to monitor other group members to collect information about their activities (e.g. vigilance, foraging or agonistic interactions) [11]. Thus, vigilance levels exhibited by group-living animals also reflect a trade-off between minimizing their risk of predation and gathering information about their social environment.

To date most of the effort devoted to studying the relationship between the group size and vigilance of prey species has been directed toward examining whether the classical group-size effect occurs at the population level [12]–[14]. The group-size effect has only recently been studied at the individual level [15]–[17]. Several studies have reported that group members may differ in their contribution to the overall group vigilance and can adopt different vigilance tactics [17]–[19]. Inter-individual differences in investment in vigilance may arise from differences between sexes [20]–[22], life history stages [19], spatial positions within the group [23] or personality types [24]. For instance, several studies have been undertaken to unravel the basis of sex differences in individual investment in vigilance [22]. The sexes may experience different vigilance-induced costs based on reproduction, physiology or personality traits, suggesting a possible sex-dependence of the vigilance tactics used to achieve the safety benefits of grouping [18], [25]. Despite the growing interest in exploring this question, little is known about the specific mechanisms underlying sex differences in vigilance, as males and females have many reasons to have evolved differences in their vigilance tactics. For instance, males trying to achieve mating or to limit the access of rivals to females should spend more time in vigilance, whereas females experiencing high costs of lactation should spend more time feeding, therefore being less vigilant. However, the presence of dependent young might lead females to increase their vigilance effort. Finally, differential vulnerability toward predators (e.g. in sexually dimorphic species), or differences in activity budgets and/or habitat use [26], [27], might also explain variation in vigilance tactic use between males and females. In addition, only a few empirical studies have examined the specific contributions of both predator detection and risk dilution and the basis of individual differences in the vigilance tactics employed by group-living animals to improve their safety [19], [28].

We studied the vigilance behaviour of adult free-ranging eastern grey kangaroos (*Macropus giganteus*) with the aim of examining whether males and females (non-reproductive or with young) adopted different vigilance tactics and varied in their reliance on predator detection and/or risk dilution as group size changed. The eastern grey kangaroo appears to be a good biological model because 1) this species shows a dynamic fission-fusion system in which individuals change groups multiple times each day and experience a large range of social situations in term of group composition and size [29] and 2) it exhibits pronounced sexual body-size dimorphism with males eventually achieving body weights more than double those reached by adult females [30]. This dimorphism might lead to a difference in vulnerability, with males suffering lower predation pressures from dingos (*Canis lupus dingo*) and feral domestic dogs (*Canis lupus familiaris*) than females, which could allow males to be less vigilant than females when in mixed-sex groups [22]. Red foxes (*Vulpes vulpes*) and wedge-tailed eagles (*Aquila audax*) are potential predators of juvenile kangaroos, which could also lead reproductive females to increase their vigilance [30].

In two earlier studies, Jarman [31] and Pays et al. [12] reported declines in mean vigilance effort with increasing group sizes in this species, consistent with the classical group-size effect. Other recent studies of eastern grey kangaroos have increased our understanding of factors affecting vigilance in this species. For instance, Pays et al. [32] reported that individuals tended to copy the vigilance activity of other group members. Moreover, individual females have been reported to vary significantly in the way that group size affects their vigilance [16]. The group-size effect may not occur in certain populations because of the result of two compensating effects: social vigilance increases whereas anti-predator vigilance decreases with group size [13]. However, individual decision making underlying variation between the sexes in vigilance tactics remains largely unexplored.

Although vigilance patterns have been extensively studied in this species, two important questions remain unsolved. What is the relative contribution of predator detection and risk dilution to anti-predator strategies? Do males and females differ in the extent to which they rely on each mechanism as a means of reducing predation risk? We addressed these questions by combining field observations of wild kangaroos with vigilance models describing the expected effects of both detection and dilution on scanning rates (number of head-up postures per minute) as group size increases [19], [33].

We based our working hypothesis on the suggested differential vulnerability between male and female eastern grey kangaroos [22], [34] and tested whether males and females feeding in the same group differed in their vigilance tactics.

## Methods

### Study Site and Animals

Field work was carried out in Sundown National Park (Queensland, Australia, 28°9′S, 151°58′E) in January–March 2009, during summer. Sundown NP is composed of a mosaic of eucalypt forest, woodland and open pastures of predominantly native species. The study area contained over 150 kangaroos. Predators of kangaroos in the study area included red foxes, wedge-tailed eagles and possibly occasional domestic and feral dogs and dingos. The study site can be characterised as semi-arid with approximately 700–800 mm of rain per year, although the rainfall is highly variable.

### Behavioural Observations

The observer (FRF) recorded behavioural sequences when animals were active, early in the morning (05.30–07.30) and late in the afternoon (17.00–19.00) when they came onto the pasture to forage. Although the study population of kangaroos was not marked for individual recognition, the observer tried to limit re-sampling of individuals by (1) studying groups from a track that crossed open paddocks, allowing him to ensure spatial independence between groups sampled in the same morning or evening session, (2) changing the direction in which he walked along the sampling track every day, (3) ensuring that no individuals had moved between the studied groups during or between the video recordings, and (4) filming only four or five groups in a day. Although the observer was confident that no group was filmed more than once during the day, it is likely that some individuals were sampled a few times during the study. We identified a group when kangaroos maintained social and spatial cohesion during focal sampling and its most peripheral member was within 15 m of another group member [31]. No ambiguities in determining group membership were encountered in the sampled groups.

The observer collected behavioural data by videotaping (Sony DCR-HC51E, Sony Corporation, Tokyo, Japan) all members of a focal group of kangaroos for a 5 min period. An animal was considered to be vigilant when it did not move its feet and raised its head above horizontal, scanning its surroundings. No ambiguities were encountered in distinguishing vigilant from non-vigilant animals. We only sampled relatively immobile groups in which individuals stayed in the same locations during the video sequences and the main activities of all group members were foraging and vigilance.

For each focal sample, the observer determined group size, group composition (i.e. numbers of adult males, adult females and juveniles) and sex of individuals (adult male, adult female with or without pouch young or young-at-foot). A pouch young is a young that either spends all of its time in its mother’s pouch, or comes and goes from the pouch, whereas a young-at-foot no longer enters the pouch but is not yet weaned. The observer also measured each individual’s distance to cover (0–25, 26–50, 51–100, 101–200, more than 200 m) and the distance to its nearest neighbour with a range finder at the end of the focal sample. To do this, the observer measured the distance between him and each individual as well as the angle between each pair of animals in the group using a protractor and then calculated inter-individual distances using trigonometric formulas. During the video sequences, group members did not exhibit apparent inter-individual interference and/or aggression, as would be expected if there was overt competition for access to food.

From the video sequences, we extracted the scanning rate (number of vigilant acts per minute) of each individual. Scanning rate was used as a measure of vigilance in accordance with the models developed by Dehn (1991), which express the relationship between the frequency of scans and group size. In our study, we did not identify the orientation of scans (toward the center or the exterior of the group) of individuals and thus could not ascertain the functions (anti-predatory or social) of the observed vigilant bouts. In total, we collected 358 samples on adult females and 20 on adult males (supplementary information on field sampling can be found in Favreau et al. [13]).

### Vigilance Models

We explored the expected effects of both predator detection and risk dilution on scanning rates by comparing 6 different candidate models of vigilance (modified from [33]; see also [19]) predicting the relationship between individuals’ scanning rates and group size. We fitted our candidate models separately to the vigilance patterns of females with and without dependant juveniles and males to investigate differences between the sexes and between females with different reproductive status.

Table 1 presents the 6 candidates models we compared. Our first candidate model (model 1: the detection model) predicts a decrease in individual vigilance solely based on increased early collective detection of predators with increasing group size. This model assumes that grouping reduces the level of individual vigilance required to maintain a given probability of predator detection. The second candidate model (model 2: the security model) combines effects from both increased predator detection and risk dilution on the relationship between vigilance and group size and predicts the probability that an individual will survive an attack. The third candidate model (model 3: the security model with non-vigilant animals), based on the previous security model, accounts for the presence of individuals in the group which are not actively vigilant. The main difference with model 2 is that the group size used to assess the effect of risk dilution (N: as all individuals participate in the dilution of risk) is different to the group size used to calculate the probability that the group detects an approaching predator (changed to Na which represents the number of actively vigilant individuals). Here juveniles are considered to be non-functionally vigilant individuals; thus the number of actively vigilant individuals is given by (group size - number of juveniles). This model nonetheless assumes that non-functionally vigilant group members contribute to the numerical dilution of risk. The fourth candidate model (model 4: the security model with mothers accompanied by dependant juveniles) considers the proportion of females with dependant young and accounts for the probability that both mothers and their offspring would survive an attack through the dilution of risk. Dehn (1990) made the assumption that females with juveniles experience a different dilution effect than other group members because they have to ensure that both they and their juvenile escape an attack. Therefore the classical expression of dilution of risk (N-1)/N has to be modified to (N-2)/N for a female with a juvenile, where N represents the group size (see the complete model derivation in [33]). Here again juveniles do not participate in collective predator detection. Finally, we developed two additional candidate models accounting for differential vulnerability between sexes. The fifth model (model 5: the low male investment (LMI) security model), derived from model 3, assumes that 1) only females (irrespective of their reproductive status) act as efficient predator detectors as they are under a higher predation risk than males and 2) males and juveniles do not participate in the detection power of the group, with males benefiting mostly from the risk dilution effect and relying on the threat detection provided by actively vigilant females. Thus the number of actively vigilant individuals in a group is given by (group size - [number of males + juveniles]). The sixth and last candidate model (model 6: LMI security model with proportion of females accompanied by dependant juveniles) is a combination of the previous one and model 4. In this model, we added the presence of mothers accompanied by dependant juveniles.

**Table 1:** Candidate vigilance models modified from Dehn (1990).

**Candidate kangaroos' vigilance models:**
1 - Detection model
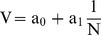
2 - Security model
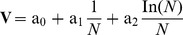
3 - Security model with non-vigilant juveniles
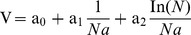
4 - Security model with mothers accompanied by dependant juveniles
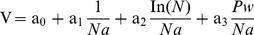
5 - Low males investment (LMI) security model
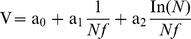
6 - Low males investment (LMI) security model accompanied by dependant juveniles
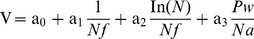

N: group size; N_a_: Number of actively vigilant individuals (N - number of juveniles); Pw: Proportion of mothers with an offspring; N_f_: Number of all actively vigilant group members (N - number of males + juveniles); ai: parameters estimates.

### Model Selection and Data Analysis

We selected the model that best explained the vigilance data using the Akaike’s information criterion (AIC) [35]. All candidate models were compared according to the AIC statistic and ranked based on their normalized Akaike weights (AICw), where the best-fitting model had the largest AICw and the smallest AIC [35]. Because of our relatively small sample size (especially for males and females with dependant juveniles) we selected our models using the AICc [36]. When ΔAICc, the difference in AICc values between two candidate models, was lower than 2, revealing a level of uncertainty surrounding these two closely competing models, we arbitrary selected the model with the lowest AICc value as the best candidate model. We obtained the AICc, ΔAICc, AICw values using the aictab function located in the AICcmodavg package in R 2.13.0 (R Development Core Team, 2011).

We ran mixed models using maximum likelihood methods (ML) to fit the set of candidate models to the vigilance data. We included each group identity within a specific observation sequence as a random effect. The random effect structure allows us to control for the effect of the group as multiple individuals from the same group were sampled during one observation session. As kangaroos were not individually marked for direct identification, we were not able to control for potential repeated measures on the same individual across different observation sessions. Non-independence of repeated measures on the same individual might affect our results. The low repeatability of scanning rate reported in bighorn sheep *(Ovis canadensis* [19] ) and in yellow-bellied marmots (*Marmota flaviventris* [37]) however, suggests that individual variation should not be a problem in our analyses. We also included the distances from the focal individual to the nearest protective cover and to the nearest group member in all models as fixed effects. Finally, all the best-fitting models selected were compared to a null model with the same random effect structure but this time only including distances to cover and to the nearest neighbour as fixed effects, using a log-likelihood ratio test.

## Results

**Figure 1:**
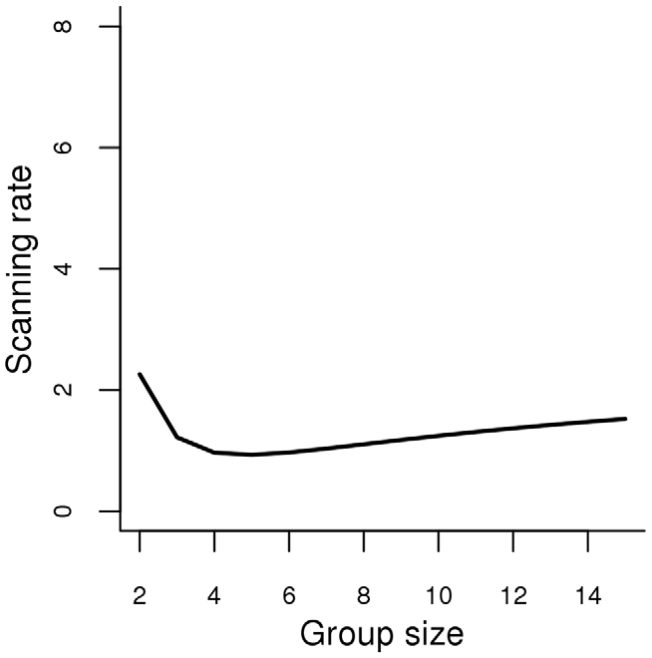
Changes in scanning rates (vigilant acts per minute) as a function of group size for mothers according to the security model.

Our results revealed that the best candidate model explaining the vigilance pattern of females accompanied by dependant juveniles was the security model (model 2) (Figure 1). The ΔAICc value between the model with the lowest AICc value (model 2) and the candidate model with the second lowest AICc value (model 5: LMI security model) was greater than 2 (ΔAICc = 4.14) (see Tables 2 and 3). Therefore we considered that the security model was the best-fitting model of our set of candidate models for females with juveniles.

**Table 2:** Model selection procedure using AICc statistics.

a)	Females accompanied by dependant juveniles			AICc	ΔAICc	AICcw	k	Observations
	**1**	**Model 2**	**Security model**	94.62	0.00	0.69	5	62
	2	Model 5	LMI security model	98.77	4.14	0.08	5	62
	3	Model 6	LMI security model with mothers with juveniles	98.85	4.23	0.08	6	61
	4	Model 4	Security model with mothers with juveniles	99.55	4.92	0.06	6	61
	5	Model 1	Detection model	99.75	5.12	0.05	4	63
	6	Model 3	Security model with non-vigilant juveniles	101.09	6.46	0.02	5	62
**b)**	**Non-reproductive females**							
	**1**	**Model 4**	**Security model with mothers with juveniles**	420.18	0.00	0.36	6	286
	2	Model 3	Security model with non-vigilant juveniles	421.00	0.83	0.24	5	287
	3	Model 2	Security model	421.49	1.31	0.19	5	287
	4	Model 6	LMI security model with mothers with juveniles	423.20	3.02	0.08	6	286
	5	Model 1	Detection model	423.44	3.26	0.07	4	288
	6	Model 5	LMI security model	424.43	4.25	0.04	5	287
**c)**	**Males**							
	1	**Model 6**	**LMI security model with mothers with juveniles**	19.78	0.00	0.35	6	15
	2	**Model 2**	**Security model**	19.83	0.05	0.33	5	16
	3	Model 4	Security model with mothers with juveniles	21.85	2.07	0.12	4	17
	4	Model 1	Detection model	22.18	2.39	0.10	6	15
	5	Model 3	Security model with non-vigilant juveniles	22.92	3.16	0.07	5	16
	6	Model 5	LMI security model	26.24	6.46	0.01	5	16

The best-fitting model for each category is presented in bold.

**Table 3:** Parameters estimates for the selected best-fitting candidate models for a) females with dependant juveniles, b) females without juvenile and c) males.

Parameter estimates						a0	a1	a2	a3
**a) Females accompanied** **by dependant juveniles**									
Model 2 - Security model						1.44	5.05	−8.07	−
**b) Non-reproductive females**									
Model 4 - Security model with mothers accompanied by juveniles						−0.24	0.17	0.48	0.57
**c) Males**									
Model 6 - LMI security model with mothers accompanied by juveniles						−0.94	−0.52	3.20	−12.16
or									
Model 2 - Security model						−0.94	−1.36	3.80	−

**Figure 2:**
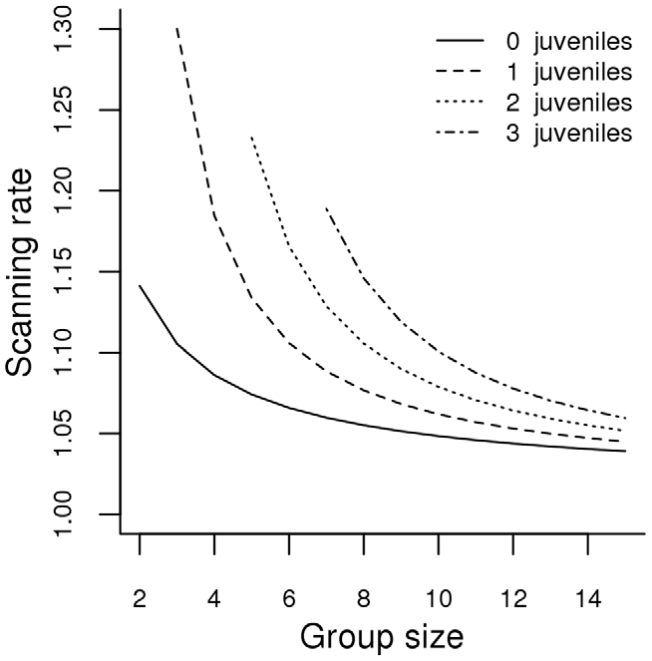
Changes in scanning rates (vigilant acts per minute) as a function of group size and number of juveniles for barren females according to model 4 (Security model with mothers accompanied by juveniles).

When considering vigilance data for non-reproductive female kangaroos, the model with the lowest AICc value was the security model that accounted for the presence of mothers with juveniles (model 4) (Figure 2). However, the security model with non-vigilant juveniles (model 3) and the security model (model 2) also appeared to fit the vigilance data for non-reproductive females relatively well (Tables 2 and 3). Despite the level of uncertainty surrounding these three competing models, we considered the security model including females with juveniles (model 4), the model with the lowest AICc value, as the best-fitting model.

**Figure 3:**
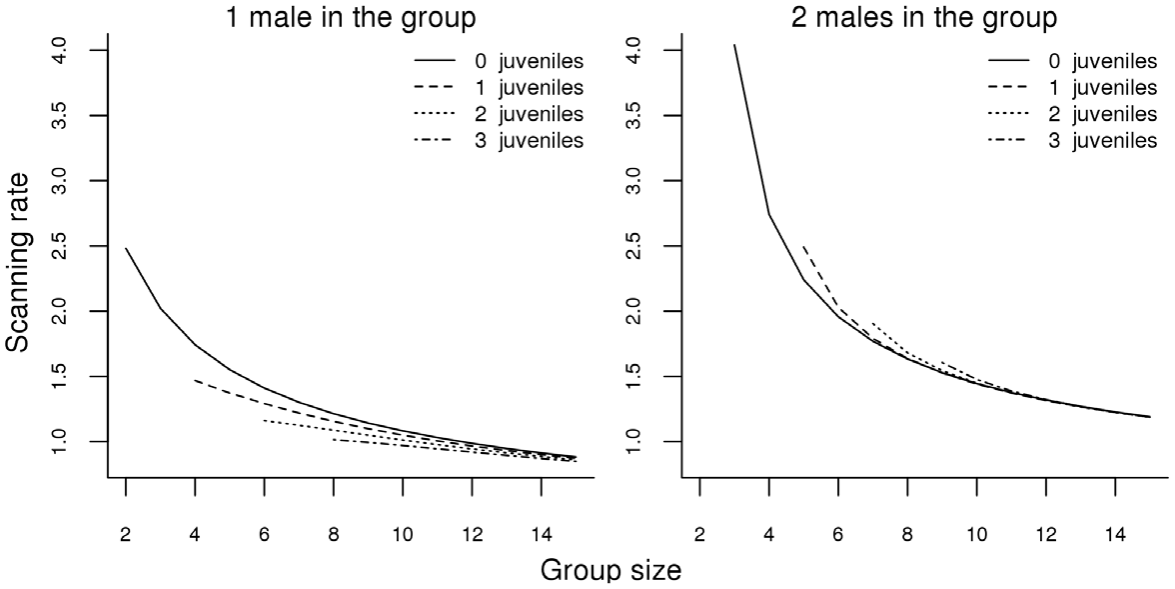
Changes in scanning rates (vigilant acts per minute) as a function of group size, number of juveniles and number of males in the group for adult males according to model 6 (LMI security model with mothers accompanied by juveniles).

For the vigilance data for males, the LMI security model accounting for mothers with juveniles (model 6) had the lowest AICc value (Figure 3). However, the difference in AICc values between this and the second best-fitting model, the security model (model 2), was very small (ΔAICc model 6 vs. model 2 = 0.05) (Tables 2 and 3). Due to the high level of uncertainty surrounding these two candidate models, we could not arbitrary select the model with the lowest AICc value as the best candidate model and we interpret male vigilance tactic use in the light of these two best-fitting models.

We found that all the selected models fitted the vigilance data significantly better than did the null model (Table 4).

**Table 4:** Comparison between the best-fitting candidate models and a null model.

					Log-likelihood ratio test	df	*P* value
**Best fitting model vs. null model**							
a) Females accompanied by dependant juveniles					11.299	2	0.003
b) Non-reproductive females					8.621	3	0.034
c) Males					12.222	3	0.006

## Discussion

Although the classical group-size effect on vigilance has previously been reported for the eastern grey kangaroos [12], [31], our results revealed some subtle differences in the vigilance strategies used by the sexes. We found differences between the vigilance strategies used by reproductive and non-reproductive females, and between those used by non-reproductive females and adult males. Although female kangaroos relied on two distinct vigilance tactics depending on their reproductive status, we did not have clear evidence of the use of different strategies by adult males and females with young, perhaps due to small sample sizes for males. Two candidate models were found to explain the vigilance tactics used by males equally well: the LMI security model accounting for mothers with juveniles and the security model. We discuss this point in view of previous results published on this topic and potential differences between the sexes that might have influenced the evolution of their vigilance tactics.

### Differences Among Females

Reproductive and non-reproductive females both relied on a combination of predator detection and risk dilution, but the latter adjusted their level of individual vigilance according to the proportion of females accompanied by juveniles within their group. Why do females differ in their vigilance tactic use? One of the main explanations is that mothers and non-mothers experience different costs of vigilance based on reproductive, physiological or nutritional trade-offs. Through experimental manipulation of reproduction in free-ranging eastern grey kangaroos using a fertility control agent, Cripps et al. [38] demonstrated that, although reproductive females did not reduce the time spent in anti-predator vigilance, females altered their behaviour in direct response to the energetic demands of reproduction. Reproductive females increased their bite rates, and thus food intake, when the energetic demands of lactation were highest. Reproduction is expected to be very costly in female eastern grey kangaroos because of their reproductive patterns [39]. At a very underdeveloped stage, a young born weighing around one gram finds its way into the pouch, attaches permanently to a nipple and continues to develop until four or five months, at which point the juvenile starts to leave the pouch for short periods of time. It is only around 10.5 months that the juvenile leaves the pouch permanently; this corresponds to the time when the mother gives birth again. The young-at-foot continues to nurse for approximately six months. Therefore, for about six months a reproductive female simultaneously provides milk to a pouch-young and a young-at-foot. Consequently many adult females invest continuously in lactation as well as devoting attention to dependent young outside the pouch.

In semi-arid environments, where food availability and quality change dramatically due to unreliable rainfall, kangaroos’ intake rates have been found to vary with vegetation biomass [40]. Under such environmental conditions, reproductive females may face a severe trade-off between time invested in meeting their nutritional requirements and those of their offspring versus other fitness-enhancing activities such as social or anti-predator vigilance. In contrast, non-reproductive females, as well as adult males, are free from such parental investment.

In an early attempt to separate the effect of the two mechanisms (improved predator detection versus risk dilution) on vigilance in a species with strong sexual segregation, Rieucau and Martin [19] found that female bighorn sheep (*O. canadensis*) rely on two distinct anti-predator tactics as the size of their group increases, depending on their reproductive status. Lactating females decreased their vigilance due to increased predator detection while non-reproductive bighorn sheep ewes decreased their individual vigilance as a function of the proportion of mothers accompanied by lambs, apparently exploiting the extra vigilance of lactating ewes. Interestingly, the similarity between bighorn sheep and eastern grey kangaroos in the differential use of vigilance tactics by females, depending on their reproductive states, suggests that the investment in vigilance of same sex group members can be unequal because some individuals exploit the supplemental predator detection effort provided by others. In light of this result, it thus appears important to take into account the possible presence of “cheaters” within a group [19] when exploring collective vigilance to clearly understand how individuals’ safety is achieved in gregarious animal species.

### Between-sex Differences

We found that two candidate models explained males’ vigilance tactic use equally well: the LMI security model accounting for mothers with juveniles (model 6) and the security model (model 2). Due to the level of uncertainty surrounding the selection of these two models care must be taken when interpreting our results. The two best candidate models both combined risk dilution and collective detection but only the former (which had a marginally lower AICc value) considers that only females with juveniles act as efficient predator detectors and that males and juveniles do not participate in the detection. This may suggest that males rely on predator detection and dilution of risk (as did females – the security model) but may also adjust their vigilance effort according to the proportion of reproductive females within a group. The small number of males sampled during this study cannot allow us to clearly distinguish between these two models. Further studies are thus needed to firmly ascertain whether male eastern grey kangaroos use a different vigilance tactic than females when foraging in mixed-sex groups and if they take advantage of the extra-vigilance effort provided by females with juveniles (as possibly suggested by model 6).

Differential vulnerability may cause sex-dependent vigilance tactic use in this dimorphic species. Adult males, which suffer a low predation pressure due to their large body sizes, may reduce their individual vigilance effort when surrounded by female group mates for whom predation risk is higher because of their smaller body sizes [22]. Childress and Lung [21] showed that female elk (*Cervus elaphus*) with calves are preferentially targeted by wolves (*Canis lupis*) and are consequently the more vigilant age-sex class. Although grouping is generally thought to reduce the likelihood of being killed during an attack, the presence of males that do little to contribute to the anti-predator detection power of the group and are less targeted by predators may cause additional security costs to females. Pays and Jarman [22] previously reported that males eastern grey kangaroos were individually less vigilant than females when groups were composed of both sexes. Moreover, they found that the vigilance of females was not affected by the presence of males within groups, suggesting that females did not perceive males as taking part in predator scanning or acting as effective dilution agents.

Unfortunately we lack information about the direction of vigilance scans in our study and therefore we cannot separate the different functions of vigilance (anti-predator or social) for each sex category. The set of candidate models used in this study assumes that vigilance is only driven by anti-predator considerations. Previous studies in eastern grey kangaroos have reported that females in single sex groups spend between 20% to 30% of their vigilant time in social vigilance [13]. As males experience lower predation risk, it is reasonable to assume that they can spend more time in social vigilance than females. However, we cannot estimate from our data the proportion of time that males devoted to monitoring conspecifics (females or rivals) when in mixed-sex groups. Males should watch for oestrous females to try to attain copulations and defend oestrous females against other male rivals. In order to better understand the causes of the differences in vigilance levels and tactics used between males and females, further effort has to be directed to the development of models that predict how social vigilance may affect the observed vigilance patterns.

We hope that our results will encourage researchers to pursue the investigation of differences among group members in the vigilance tactics employed and the basis of such inter-individual differences. We should now reexamine the classical view of the safety advantages providing by grouping to provide a better understanding of the mechanisms and functions of gregariousness. A further achievement would be the inclusion of social vigilance into the different candidate models to accurately predict vigilance patterns of group living animals.

## References

[1] Krebs JR, Davies NB (1993) An introduction to behavioural ecology. Oxford: Blackwell Science.

[2] Caro T (2005) Antipredator defenses in birds and mammals: University of Chicago Press.

[3] Lima SL (1995) Back to the basics of anti-predatory vigilance: the group-size effect. Animal Behaviour 49: 11–20.

[4] Pulliam HR (1973) On the advantages of flocking. Journal of Theoretical Biology 38: 419–422. 4734745 10.1016/0022-5193(73)90184-7

[5] Pulliam HR, Pyke GH, Caraco T (1982) The scanning behavior of juncos: A game-theoretical approach. Journal of Theoretical Biology 95: 89–103.

[6] Lima SL, Dill LM (1990) Behavioral decisions made under the risk of predation: a review and prospectus. Canadian Journal of Zoology 68: 619–640.

[7] Lima SL (1998) Nonlethal effects in the ecology of predator-prey interactions. BioScience 48: 25–34.

[8] Lima SL, Zollner PA, Bednekoff PA (1999) Predation, scramble competition, and the vigilance group size effect in dark-eyed juncos (*Junco hyemalis)* . Behavioral Ecology and Sociobiology 46: 110–116.

[9] Duncan P, Foose TJ, Gordon IJ, Gakahu CG, Lloyd M (1990) Comparative nutrient extraction from forages by grazing bovids and equids: a test of the nutritional model of equid/bovid competition and coexistence. Oecologia 84: 411–418. 28313034 10.1007/BF00329768

[10] Fortin D, Boyce MS, Merrill EH, Fryxell JM (2004) Foraging costs of vigilance in large mammalian herbivores. Oikos 107: 172–180.

[11] Fernández-Juricic E, Kacelnik A (2004) Information transfer and gain in flocks: the effects of quality and quantity of social information at different neighbour distances. Behavioral Ecology and Sociobiology 55: 502–511.

[12] Pays O, Renaud P-C, Loisel P, Petit M, Gerard J-F, et al (2007) Prey synchronize their vigilant behaviour with other group members. Proceedings of the Royal Society B: Biological Sciences 274: 1287–1291. 17341457 10.1098/rspb.2006.0204 PMC2176174

[13] Favreau F-R, Goldizen AW, Pays O (2010) Interactions among social monitoring, anti-predator vigilance and group size in eastern grey kangaroos. Proceedings of the Royal Society B: Biological Sciences 277: 2089–2095. 20219737 10.1098/rspb.2009.2337 PMC2880096

[14] Michelena P, Deneubourg J-L (2011) How group size affects vigilance dynamics and time allocation patterns: The key role of imitation and tempo. PLoS ONE 6: e18631. 21525987 10.1371/journal.pone.0018631 PMC3078120

[15] Beauchamp G (2008) What is the magnitude of the group-size effect on vigilance? Behavioral Ecology 19: 1361–1368.

[16] Carter A, Pays O, Goldizen A (2009) Individual variation in the relationship between vigilance and group size in eastern grey kangaroos. Behavioral Ecology and Sociobiology 64: 237–245.

[17] Rieucau G, Morand-Ferron J, Giraldeau L-A (2010) Group size effect in nutmeg mannikin: between-individuals behavioral differences but same plasticity. Behavioral Ecology 21: 684–689.

[18] Lung MA, Childress MJ (2007) The influence of conspecifics and predation risk on the vigilance of elk (*Cervus elaphus*) in Yellowstone National Park. Behavioral Ecology 18: 12–20.

[19] Rieucau G, Martin JGA (2008) Many eyes or many ewes: vigilance tactics in female bighorn sheep (*Ovis canadensis*) vary according to reproductive status. Oikos 117: 501–506.

[20] Clare D F (1990) Why do hunting cheetahs prefer male gazelles? Animal Behaviour 40: 837–845.

[21] Childress MJ, Lung MA (2003) Predation risk, gender and the group size effect: does elk vigilance depend upon the behaviour of conspecifics? Animal Behaviour 66: 389–398.

[22] Pays O, Jarman P (2008) Does sex affect both individual and collective vigilance in social mammalian herbivores: the case of the eastern grey kangaroo? Behavioral Ecology and Sociobiology 62: 757–767.

[23] Blanchard P, Sabatier R, Fritz H (2008) Within-group spatial position and vigilance: a role also for competition? The case of impalas (*Aepyceros melampus*) with a controlled food supply. Behavioral Ecology and Sociobiology 62: 1863–1868.

[24] Quinn JL, Cresswell W (2005) Personality, anti-predation behaviour and behavioural plasticity in the chaffinch *Fringilla coelebs* . Behaviour 142: 1377–1402.

[25] López P, Hawlena D, Polo V, Amo L, Martín J (2005) Sources of individual shy–bold variations in antipredator behaviour of male Iberian rock lizards. Animal Behaviour 69: 1–9.

[26] Ruckstuhl KE, Neuhaus P (2002) Sexual segregation in ungulates: a comparative test of three hypotheses. Biological Reviews 77: 77–96. 11911375 10.1017/s1464793101005814

[27] Michelena P, Bouquet PM, Dissac A, Fourcassie V, Lauga J, et al (2004) An experimental test of hypotheses explaining social segregation in dimorphic ungulates. Animal Behaviour 68: 1371–1380.

[28] Fairbanks B, Dobson FS (2007) Mechanisms of the group-size effect on vigilance in Columbian ground squirrels: dilution versus detection. Animal Behaviour 73: 115–123.

[29] Carter AJ, Macdonald SL, Thomson VA, Goldizen AW (2009) Structured association patterns and their energetic benefits in female eastern grey kangaroos, Macropus giganteus. Animal Behaviour 77: 839–846.

[30] Jarman PJ (1989) Sexual dimorphism in Macropodoidea. In: Grigg G JP, Hume I editor. Kangaroos, wallabies and rat-kangaroos: Surrey Beatty, Chipping Norton. 433–447.

[31] Peter J J (1987) Group size and activity in eastern grey kangaroos. Animal Behaviour 35: 1044–1050.

[32] Pays O, Goulard M, Blomberg SP, Goldizen AW, Sirot E, et al (2009) The effect of social facilitation on vigilance in the eastern gray kangaroo, Macropus giganteus. Behavioral Ecology 20: 469–477.

[33] Dehn M (1990) Vigilance for predators: detection and dilution effects. Behavioral Ecology and Sociobiology 26: 337–342.

[34] Colagross A, Cockburn A (1993) Vigilance and Grouping in the Eastern Gray Kangaroo, Macropus giganteus. Australian Journal of Zoology 41: 325–334.

[35] Burnham KP, Anderson DR (2002) Model selection and multi-model inference: a practical information-theoretic approach: Springer.

[36] Hurvich CM, Tsai C-L (1989) Regression and time series model selection in small samples. Biometrika 76: 297–307.

[37] Blumstein DT, Lea AJ, Olson LE, Martin JGA (2010) Heritability of anti-predatory traits: vigilance and locomotor performance in marmots. Journal of Evolutionary Biology 23: 879–887. 20298440 10.1111/j.1420-9101.2010.01967.x

[38] Cripps JK, Wilson ME, Elgar MA, Coulson G (2011) Experimental manipulation of fertility reveals potential lactation costs in a free-ranging marsupial. Biology Letters 7: 859–862. 21733874 10.1098/rsbl.2011.0526 PMC3210684

[39] Jarman PJ, Southwell CJ (1986) Grouping, associations and reproductive strategies in eastern grey kangaroos. In: Rubenstein DI WR, editor. Ecological aspects of social evolution: birds and mammals: Princeton University Press, Princeton, New Jersey,. 399–428.

[40] Short J (1985) The functional response of kangaroos, sheep and rabbits in an arid grazing system. Journal of Applied Ecology 22: 435–447.

