# Correction: Investigating Differences in Vigilance Tactic Use within and between the Sexes in Eastern Grey Kangaroos

**DOI:** 10.1371/annotation/e954af39-a5bd-4bbd-aa64-b6a53edb4557

**Published:** 2012-10-31

**Authors:** Guillaume Rieucau, Pierrick Blanchard, Julien G. A. Martin, François-René Favreau, Anne W. Goldizen, Olivier Pays

There was an error in the Funding section. The correct funding information is as follows: GR was financially supported by a Fyssen Foundation postdoctoral fellowship and by a postdoctoral fellowship from the Institute of Marine Research, Bergen, Norway. This work was also partially supported by the Norwegian Research Council (grant 204229/F20). GR and PB are at the Laboratoire Evolution et Diversité Biologique (EDB) part of the "Laboratoire d'Excellence" (LABEX) entitled TULIP (ANR -10-LABX-41). JM was funded by an Fonds de recherche du Québec Nature et technologies (FQRNT) fellowship and an National Science Foundation - Long-Term Research in Environmental Biology (NSF-LTREB) grant. AWG and F-RF were supported by research funds from the University of Queensland. The funders had no role in study design, data collection and analysis, decision to publish, or preparation of the manuscript. 

